# Daily Activities Related to Mobile Cognitive Performance in Middle-Aged and Older Adults: An Ecological Momentary Cognitive Assessment Study

**DOI:** 10.2196/19579

**Published:** 2020-09-24

**Authors:** Laura M Campbell, Emily W Paolillo, Anne Heaton, Bin Tang, Colin A Depp, Eric Granholm, Robert K Heaton, Joel Swendsen, David J Moore, Raeanne C Moore

**Affiliations:** 1 SDSU/UC San Diego Joint Doctoral Program in Clinical Psychology San Diego, CA United States; 2 Department of Psychiatry University of California San Diego San Diego, CA United States; 3 VA San Diego Healthcare System San Diego, CA United States; 4 CNRS UMR University of Bordeaux Bordeaux France; 5 National Center for Scientific Research Ecole Pratique des Hautes Etudes, PSL Research University Paris France

**Keywords:** ecological momentary assessment, daily functioning, telemedicine, digital health, neuropsychological test, cognition, HIV, aging, mobile phone

## Abstract

**Background:**

Daily activities have been associated with neurocognitive performance. However, much of this research has used in-person neuropsychological testing that requires participants to travel to a laboratory or clinic, which may not always be feasible and does not allow for the examination of real-time relationships between cognition and behavior. Thus, there is a need to understand the real-time relationship between activities in the real world and neurocognitive functioning to improve tracking of symptoms or disease states and aid in the early identification of neurocognitive deficits among at-risk individuals.

**Objective:**

We used a smartphone-based ecological momentary cognitive assessment (EMCA) platform to examine real-time relationships between daily activities and neurocognitive performance (executive functioning and verbal learning) in the everyday environment of middle-aged and older adults with and without HIV.

**Methods:**

A total of 103 adults aged 50-74 years (67 persons with HIV; mean age 59 years, SD 6.4) were recruited from the University of California, San Diego HIV Neurobehavioral Research Program and the San Diego community. Participants completed our EMCA protocol for 14 days. Participants reported their current daily activities 4 times per day; following 2 of the 4 daily ecological momentary assessment (EMA) surveys, participants were administered the mobile Color-Word Interference Test (mCWIT) and mobile Verbal Learning Test (mVLT), each once per day. Activities were categorized into cognitively stimulating activities, passive leisure activities, and instrumental activities of daily living (IADLs). We used multilevel modeling to examine the same-survey and lagged within-person and between-person effects of each activity type on mobile cognitive performance.

**Results:**

On average, participants completed 91% of the EMA surveys, 85% of the mCWIT trials, and 80% of the mVLT trials, and they reported engaging in cognitively stimulating activities on 17% of surveys, passive leisure activities on 33% of surveys, and IADLs on 20% of surveys. Adherence and activity percentages did not differ by HIV status. Within-persons, engagement in cognitively stimulating activities was associated with better mCWIT performance (β=−1.12; *P*=.007), whereas engagement in passive leisure activities was associated with worse mCWIT performance (β=.94; *P=*.005). There were no lagged associations. At the aggregate between-person level, a greater percentage of time spent in cognitively stimulating activities was associated with better mean mVLT performance (β=.07; *P=*.02), whereas a greater percentage of time spent in passive leisure activities was associated with worse mean mVLT performance (β=−.07; *P=*.01). IADLs were not associated with mCWIT or mVLT performance.

**Conclusions:**

Smartphones present unique opportunities for assessing neurocognitive performance and behavior in middle-aged and older adults’ own environment. Measurement of cognition and daily functioning outside of clinical settings may generate novel insights on the dynamic association of daily behaviors and neurocognitive performance and may add new dimensions to understanding the complexity of human behavior.

## Introduction

### Background

Traditional neuropsychological testing provides a *snapshot* of a patient’s neurocognitive functioning at one time point in an optimal, controlled environment (ie, without distractions). This assessment method allows neuropsychologists to make empirically based judgments about a number of critical patient factors (eg, neurocognitive impairment status and likely etiology of deficits). However, there are several limitations associated with traditional laboratory-based neuropsychological assessments. First, traditional testing requires in-person, face-to-face contact. The limitations of this are more salient now than ever before owing to the need for social distancing because of the COVID-19 pandemic [1]. However, even before the COVID-19 pandemic, there were several barriers associated with in-person neuropsychological testing. For example, it is difficult for individuals with limited access to transportation or those who live in rural areas to travel to clinics and/or participate in research. Second, although one goal of neuropsychological assessment is often to make judgments about everyday functioning, the connection between performance on neuropsychological testing and neurocognitive functioning in daily life is imperfect [2]—people do not always perform consistently in an optimal manner in everyday life. In other words, a person’s neurocognitive capacity, as demonstrated in a neuropsychological testing room, may not predict their performance in everyday tasks that take place in a more distracting, real-world environment [3,4].

Now, more than ever, we need to leverage digital health tools to improve upon assessment of cognition [5,6]. One feasible solution to the limitations of traditional neuropsychological testing is the utilization of mobile cognitive testing. This burgeoning digital health assessment method involves objective cognitive tests that are self-administered through a smartphone, tablet, or other mobile device. Thus, mobile cognitive testing both reduces the need for in-person visits and allows for repeated assessment of neurocognitive functioning in an individual’s natural environment [7,8]. Therefore, this methodology may be particularly useful for detecting neurocognitive decline earlier, given that performance on tests can be easily tracked over time and repeatedly compared with one’s own previous performance. Moreover, mobile cognitive testing may be more ecologically valid and thus help to better understand subtle changes in neurocognitive functioning as they occur in participants’ own environments. Despite some technological challenges (eg, standardizing stimuli and response latencies across different software and hardware platforms) [9], mobile cognitive testing has been found to be feasible and valid among various clinical [7,10,11] and nonclinical populations [7,10-12].

Mobile cognitive testing may be a particularly important tool for a better understanding of the relationship between real-world neurocognitive and everyday functioning. In the context of a clinical neuropsychological evaluation, the relationship between cognition and everyday functioning is often considered to be unidirectional, as neuropsychologists use neurocognitive test data to predict everyday functioning outcomes. However, a bidirectional relationship between certain everyday activities and neurocognitive functioning has been demonstrated in older adults [13]. For example, time spent in passive leisure activities (eg, watching television) has been shown to be inversely related to cross-sectional neurocognitive performance and positively related to the likelihood of a neurocognitive decline in subsequent years [14-18]. Conversely, studies have also demonstrated a positive relationship between time spent in cognitively, physically, and/or socially stimulating activities and neurocognitive functioning in older adults [19] and persons with HIV [20]. The existing literature examining relationships between everyday activities and cognition, however, is limited in several ways. Self-report retrospective measures may be inaccurate because of recall error and response biases [21,22], whereas performance-based measures of everyday functioning are limited in their ability to be administered frequently (eg, need for in-person clinic visits, practice effects) and were developed to measure functional capacity, that is, whether a person has the capacity to function independently. However, many are not validated as tools to assess functional performance, that is, how people actually function in their home environments [23]. In addition, most studies have only examined between-person effects (eg, baseline activities predicting change in cognition over time) [17]. Thus, little is known about the possible acute and dynamic relationships between everyday activities and cognition within individuals.

Coupling mobile cognitive testing with ecological momentary assessment (EMA; ie, repeated self-report assessment of in-the-moment feelings, behaviors, and contexts) affords the opportunity to examine real-world, real-time relationships between daily activities and neurocognitive performance. For example, in the only study to date (to our knowledge) that has examined the within-person relationship between daily activities and cognition using EMA and mobile cognitive testing, Allard et al [24] found that engagement in cognitively stimulating activities was associated with better semantic memory performance later in the day in a sample of older adults. However, participation in passive leisure activities was not significantly associated with any differences in performance on the mobile test of semantic memory [24]. This study serves as an example of how understanding the dynamic relationships between daily life activities and cognition could lead to improved symptoms or disease tracking or just-in-time adaptive interventions to promote optimal neurocognitive functioning, when desired, using digital health technologies. Such interventions may be important for older adults [25] and persons with HIV [26,27], particularly older persons with HIV, given the higher rates of neurocognitive impairment compared with the general population.

### Objectives

In light of these questions, this study used a smartphone-based ecological momentary cognitive assessment (EMCA) platform (ie, a custom-built integrated EMA and mobile cognitive testing platform) to examine the real-world relationships between daily activities (ie, cognitively stimulating activities, passive leisure activities, and instrumental activities of daily living [IADLs]) and neurocognitive performance (ie, executive functioning and learning) among older persons with and without HIV. The first aim of this study is to examine (1) the same-survey, within-person relationships between reported activities and mobile cognitive performance and (2) the between-person effect of the percentage of surveys in which the activity was endorsed with mean mobile cognitive test performance. Accounting for between-person effects allows for differentiation between whether an activity is associated with true changes and/or fluctuations in cognition or whether more total time spent in an activity, in general, tends to be associated with a higher or lower level of cognition. The second aim is to examine the temporal ordering of effects, with activity engaged in at one survey predicting cognition at the same-day next survey, administered approximately 3 to 4 hours later (ie, lagged analyses). We hypothesized that in all analyses, cognitively stimulating activities would be associated with better mobile cognitive performance, and passive leisure activities would be associated with worse mobile cognitive performance. In addition, we hypothesized that engagement in IADLs would be associated with better mobile cognitive performance, but the effect would be weaker than that observed with cognitively stimulating activities. Given that potential differences in observed effects by HIV serostatus would be likely because of differences in a combination of sociodemographic and/or environmental factors rather than HIV itself, we accounted for HIV status but did not specifically examine any HIV interactions.

## Methods

### Participants

A total of 67 community-dwelling persons with HIV and 36 HIV-negative middle-aged and older adults, aged 50 to 74 years, were included in this National Institute of Mental Health (NIMH)–funded study at the HIV Neurobehavioral Research Program (HNRP) at the University of California, San Diego (UCSD) between 2016 and 2019. Participants were recruited from the participant pool at the HNRP or through the community (eg, HIV clinics, flyers, and community centers). Inclusion criteria for the study were being aged ≥50 years, the ability to provide written informed consent, and being fluent in English. Exclusion criteria for the study were self-reported histories of serious mental illness (eg, schizophrenia and bipolar disorder), non-HIV neurological disorder (eg, stroke), head injury with loss of consciousness for ≥30 min, or indication of a severe learning disability (as indicated by a score of <70 on the Wide Range Achievement Test, fourth edition Reading Subtest [WRAT-4]). Participants with a positive urine toxicology test (with the exception of marijuana) or alcohol breathalyzer on the day of the in-person visit were rescheduled. All procedures were approved by UCSD’s Institutional Review Board before protocol implementation, and all participants demonstrated decisional capacity [28] and provided written informed consent.

### Measures and Procedures

#### Laboratory Visits

Participants completed an initial in-person baseline visit that included a tutorial on the EMCA portion of the study and a comprehensive neuromedical and neurobehavioral assessment. Participants were given a Samsung Galaxy S 4.2 YP-GI1 8GB smartphone with a 4G Android operating system (OS) for the duration of the study. The Galaxy Player 4.2 has a 4.2“ IPS (in-plane switching) display at 800×480 resolution, 1 GHz processor, using Android 2.3.6 Gingerbread OS. Participants were provided an individualized, face-to-face 20- to 30-min tutorial with a research associate on how to complete EMA surveys and mobile cognitive tests and were given a smartphone operating manual to take home.

Psychiatric and substance use disorders in Table 1 were determined via a computer-assisted structured interview, that is, the Composite International Diagnostic Interview [29]. In-person neurocognitive functioning in Table 1 was determined by comprehensive neuropsychological assessment (previously described in detail by Heaton et al [27]; see Multimedia Appendix 1 for specific neuropsychological tests), and neurocognitive domain scaled scores (mean 10, SD 3) that adjust for practice effects were generated [30]. Impairment was determined using a global deficit score [31] of ≥0.5, and premorbid verbal IQ was measured via the WRAT-4 [32]. For all participants, HIV serostatus was confirmed with HIV/Hepatitis C virus antibody point-of-care rapid test (Miriad-MedMira) and confirmed by western blot analyses. Among persons with HIV, AIDS diagnosis, antiretroviral therapy (ART) regimen, estimated duration of HIV disease, and nadir CD4 count were obtained by self-report (unless the current CD4 count was lower than the reported nadir CD4 value). Viral load detectability (≥50 copies/mL) and the current CD4 count was measured in blood plasma.

At the end of the 14-day EMA study period, participants returned to the HNRP to deliver the study phone and completed additional neuropsychological tests and study questionnaires. Participants were compensated for both study visits. Bonus compensation (US $1 per survey) was provided for each EMA survey participants completed.

**Table 1:** Participants’ characteristics by HIV serostatus (N=103).

Participant characteristics				HIV+ (n=67)		HIV− (n=36)		*t* value or chi-square		*P* value
**Demographic variables**										
		Age (years), mean (SD)	59.3 (6.3)		59.2 (6.7)		0.0		.99	
		Sex (male), n (%)	53 (79)		20 (56)		6.3		.01	
	**Race or ethnicity, n (%)**		N/A^a^		N/A		1.3		.73	
		Non-Hispanic White	42 (63)		23 (64)					
		African American	16 (24)		6 (17)					
		Hispanic	4 (10)		6 (17)					
		Other	2 (3)		1 (3)					
	Education (years), mean (SD)		13.9 (2.4)		15.0 (2.5)		−2.1		.04	
	Employed^b^, n (%)		20 (31)		14 (40)		0.9		.35	
	**Household income^c^ (US$), n (%)**		N/A		N/A		FET^d^		.002	
		<10,000	12 (18)		6 (17)					
		10,000-34,999	45 (68)		13 (36)					
		35,000-74,999	4 (6)		8 (22)					
		≥75,000	5 (8)		9 (25)					
	Live alone, n (%)		38 (57)		11 (31)		6.6		.01	
	Smartphone ownership, n (%)		60 (90)		30 (83)		0.8		.36	
**Psychiatric comorbidities, n (%)**										
		LT^e^ MDD^f^	48 (72)		9 (25)		20.6		<.001	
		Current MDD^g,h^	11 (16)		1 (3)		FET		.05	
		LT any substance use disorder	45 (67)		17 (47)		3.9		.04	
		Current substance use disorder^h,i^	2 (3)		1 (3)		FET		.99	
**In-person neurocognitive functioning**										
		GDS^j^ impaired^c^, n (%)	18 (27)		7 (20)		0.59		.44	
		Global SS^c,k^, mean (SD)	9.2 (2.0)		9.9 (1.8)		−1.6		.11	
		Verbal SS^c^, mean (SD)	10.2 (2.7)		11.4 (2.8)		−2.0		.05	
		Executive functioning SS^c^, mean (SD)	8.9 (2.5)		9.8 (2.0)		−1.7		.10	
		Speed of information processing SS^c^, mean (SD)	9.6 (2.4)		10.4 (2.5)		−1.4		.16	
		Learning SS^c^, mean (SD)	8.3 (2.3)		9.1 (2.3)		−1.8		.07	
		Recall SS^c^, mean (SD)	8.6 (2.2)		9.3 (2.4)		−1.5		.14	
		Working memory SS^c^, mean (SD)	9.8 (2.9)		10.3 (2.8)		−0.8		.42	
		Motor SS^c^, mean (SD)	7.8 (2.7)		8.0 (2.4)		−0.4		.69	
		Premorbid verbal IQ^h,l^, mean (SD)	102 (14)		106 (16)		−1.2		.24	
**HIV characteristics**										
		AIDS^m^, n (%)	46 (70)		N/A		N/A		N/A	
		Current CD4^m^, median (IQR)	703 (550-893)		N/A		N/A		N/A	
		Nadir CD4^m^, median (IQR)	148 (33-285)		N/A		N/A		N/A	
		Duration of HIV infection (years), median (IQR)	23.8 (15.7-28.8)		N/A		N/A		N/A	
		On antiretroviral therapy, n (%)	63 (94)		N/A		N/A		N/A	
		Undetectable viral load^n^, n (%)	60 (97)		N/A		N/A		N/A	

^a^N/A: not applicable.

^b^n=100.

^c^n=102.

^d^FET: Fisher exact test.

^e^LT: lifetime.

^f^LT MDD: met criteria for major depressive disorder at any point in life.

^g^Current MDD: currently meets criteria for major depressive disorder.

^h^n=101.

^i^All current substance use disorders were marijuana use disorder.

^j^GDS: global deficit score.

^k^SS: scaled score; scaled score is based on comprehensive in-laboratory neuropsychological testing (scale score: mean 10, SD 3).

^l^Premorbid Verbal IQ was estimated using the Wide Range Achievement Test, fourth edition Reading Subtest.

^m^n=66.

^n^n=62.

#### Fourteen-Day EMA and Mobile Cognitive Testing Monitoring

Following their first in-person visit, participants completed the 14-day EMCA protocol. Participants received 4 EMA surveys per day, which occurred at pseudorandom times throughout the day (ie, spaced for a survey to occur in the morning, midday, afternoon, and evening) approximately 3 to 4 hours apart according to each participant’s sleep-wake schedule. The study phone alert sounded to signal participants to complete each survey. Once the alert sounded, participants had 10 min to start the survey, with a reminder alarm every 2 min during that period before the survey was considered missed. Participants also had the option to cancel the survey during the 10-min window or at any point during the survey. If a participant only completed part of the survey and then canceled, the data completed were saved. At the end of 2 of the 4 daily surveys, participants were prompted to complete either a mobile cognitive test of executive functioning or verbal learning. The surveys after which participants received the mobile cognitive tests were randomized to different surveys per day and were presented at the same survey per day for each participant. The mobile Color-Word Interference Test (mCWIT) and mobile Verbal Learning Test (mVLT) were not given on the same survey. The study phone’s OS was encrypted, in the event that the phone was lost or stolen, to safeguard participants’ data. Furthermore, the study phones were locked so that the survey program was the only program on the phone that could be used. Participants were provided contact information in the event that they experienced technological difficulties and were called twice during the 14-day period to assess if they had any difficulties.

##### Daily Activities

**Figure 1:**
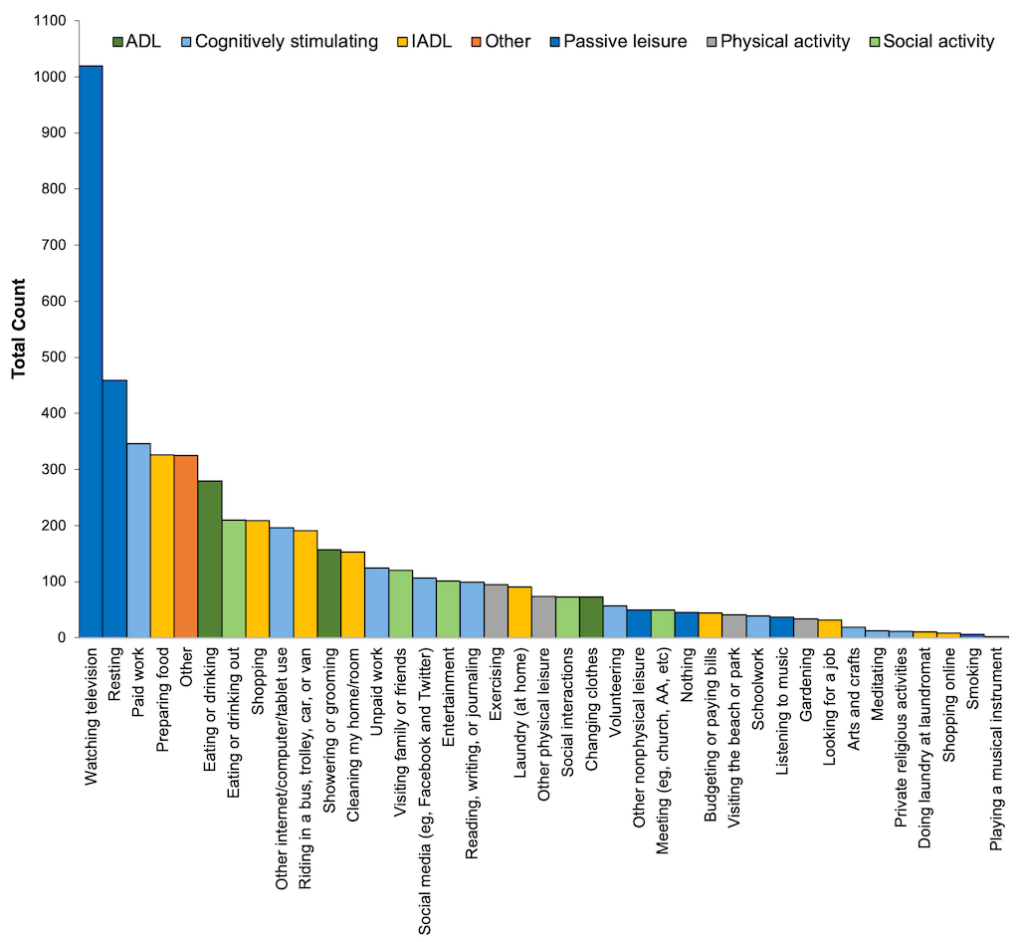
Count of reported activities from the ecological momentary assessment question “What are you doing?” Participants could only report one activity and were instructed to choose the primary activity in which they were currently participating. Participants could choose “other” if their current activity was not listed as an option. AA: alcoholics anonymous; ADL: activities of daily living; IADL: instrumental activities of daily living.

At each survey, participants were asked to report their current activity (ie, “What are you doing?”). Participants could choose 1 response from 39 options that included different activities (Figure 1), with different options available, depending on whether the participant reported being at home or not at home on a previous question. Participants were only given the option to report 1 activity in response to this item and were instructed to choose the primary activity in which they were currently participating. Activities were then categorized into cognitively stimulating activities, passive leisure activities, IADLs, activities of daily living (ADLs), physical activity, social activities, and other activities (ie, if participants endorsed *other* as their activity). Cognitively stimulating activities included working (paid or unpaid), volunteering, schoolwork, arts and crafts, meditating, playing a musical instrument, private religious activities, reading or writing or journaling, and *other internet or computer or tablet use*. Passive leisure activities included: watching television, listening to music, other nonphysical leisure, resting, smoking, social media, and nothing. IADLs included budgeting or paying bills, cleaning, doing laundry, looking for a job, preparing food, traveling (ie, riding in a bus, trolley, car, or van), and shopping. These activities were categorized by 3 authors (RM, CD, and EG) with expertise in this area of research and who reviewed the current literature. ADLs, physical activity, social activities, and *other* were each endorsed on less than 10% of surveys and were thus not examined in this study. Cognitively stimulating activities, passive leisure activities, and IADLs were dichotomized into 0 (*not doing activity*) and 1 (*doing activity*). The percentage of time spent in each activity was calculated by dividing the number of surveys in which an activity was endorsed by the total number of completed EMA surveys over the 14-day study period.

#### Mood

Depressed mood was assessed at each EMA survey, and response choices for the prompt, “I feel depressed...,” were on a 5-point scale ranging from *1=not at all* to *5=very much.* Although not the main focus of this study, the current depression rating was included as a covariate in follow-up analyses to account for mood as a possible confounding factor.

#### Mobile Cognitive Tests

Participants completed an mCWIT and mVLT once per day. The survey in which they were prompted to complete the mobile cognitive test was randomized and counterbalanced across the different times of the day for the 14-day EMA period. The mCWIT is a test of executive functioning based on the Stroop paradigm whereby people are asked to name the color of the ink in which a color-word is written when the ink color and word are incongruent. Stroop tasks have been used widely among persons with HIV [33,34]; however, there is only one other group to our knowledge that has developed a mobile Stroop-like test for use in an EMA study [11]. Participants were instructed, “Do not read the words, say the colors in which they are written” and had up to 60 seconds to complete the task. Responses were audio-recorded and scored by 2 independent raters. All discrepant scores were scored by a third rater. The outcome assessed in this study was completion time (seconds). mCWIT data were excluded from analyses for 2 participants; one participant was excluded due to colorblindness, and the other participant was excluded because they had an average of 15 of 16 errors, and therefore their data were not considered valid. The reliability and validity of the mCWIT have been described in detail elsewhere [10].

The mVLT is a test designed to assess verbal learning and recall. Although there are other groups who have developed verbal learning or memory tests for use in mobile cognitive testing studies, none have examined these among persons with HIV [8]. Participants were presented with a list of 12 semantically unrelated words to read on the smartphone for three 30-second trials. A unique list was presented each day. After each trial, participants were asked to recall words from the list and then to select *done* when they finished recalling words. Responses were audio-recorded and scored for the total number of correct responses. The outcome assessed in this study was the total number of words correctly recalled over the 3 learning trials. The mVLT was scored by 2 independent raters, and discrepant scores were reviewed by an additional rater. The association between the mVLT (mean average across 14 days) and in-laboratory Hopkins Verbal Learning Test-Revised [35] was Cohen *d*=1.09. On both tests, there was some intraindividual variability in performance across days, as expected (average SD per person mCWIT=5.2 seconds; mVLT=3.5 words). mCWIT and mVLT trials were excluded from this study if raters suspected cheating (eg, help from others), if the participant was doing something else during the test (eg, talking with others), or if the participant endorsed the use of illicit substances (ie, cocaine, methamphetamine, ecstasy, heroin, *other street drug(s),* or “prescription drugs not prescribed to me”) on any survey before taking the mobile cognitive test (within the same day). In follow-up analyses, analyses were rerun excluding surveys in which participants endorsed alcohol or marijuana use on the same survey.

### Statistical Analyses

Participant characteristics by HIV serostatus are presented in Tables 1 and 2 and were assessed via two-tailed chi-square, Fisher exact tests, and *t* tests (or nonparametric equivalent), as appropriate. Separate linear mixed-effects regressions with participant-specific random intercepts were used to evaluate the relationship between activity reported on EMA (ie, within-person effect) and neurocognitive performance (ie, mCWIT and mVLT performance) as well as the percentage of surveys in which each activity was reported and the average neurocognitive performance (ie, the between-person effect). Two sets of analyses were completed: (1) same-survey associations, with activity selected on the EMA survey as a predictor of neurocognitive performance at the same survey (ie, mCWIT and mVLT performance), and (2) lagged associations, with activity at the previous survey within the same day as a predictor of next-survey neurocognitive performance. All models controlled for HIV status and study day (1-14, centered to day 1). Age, sex, education, and race or ethnicity (non-Hispanic White vs all other race or ethnicities) were initially included in the models if they were associated with the mobile cognitive test score at *P*<.10, and were retained as covariates if they remained associated with the outcome at *P*<.10. Therefore, mCWIT analyses included age and race or ethnicity as covariates, and mVLT analyses included education as a covariate. All analyses were reexamined with depressed mood as a covariate and excluding surveys in which participants reported alcohol or marijuana use. Results were considered statistically significant at *P*<.05. R software (version 3.6.1) was used for all analyses, and the lme4 package for R was used for mixed-effects regression analyses.

**Table 2:** Ecological momentary assessment and mobile cognitive test variables by HIV serostatus (N=103).

Variable		HIV+ (n=67)	HIV− (n=36)	*t* value or chi-square	*P* value
**EMA^a^ variables, mean (SD)**					
	Number of EMA surveys completed	91 (9.0)	90 (8.5)	0.3	.80
	Percent of cognitively stimulating activities	16 (15.5)	19 (13.2)	−1.0	.09
	Percent of passive leisure activities	35 (17.2)	29 (14.0)	1.7	.15
	Percent of IADLs^b^	19 (8.1)	22 (9.0)	−1.7	.08
**Mobile cognitive testing variables**					
	Average mCWIT^c^ total seconds, mean (SD)	24.0 (6.7)	21.6 (4.2)	2.2	.03
	Percent mCWIT completed, mean % (SD)	84 (18.5)	84 (20.0)	−0.1	.95
	Average mVLT^d^ total words, mean (SD)	19.7 (4.9)	21.1 (3.8)	−1.4	.15
	Percent mVLT completed, mean % (SD)	79 (18.9)	82 (13.3)	−0.9	.39

^a^EMA: ecological momentary assessment.

^b^IADLs: instrumental activities of daily living.

^c^mCWIT: mobile Color-Word Interference Test.

^d^mVLT: mobile Verbal Learning Test.

## Results

### Sample Characteristics

Participants’ demographic and clinical characteristics by HIV serostatus are presented in Table 1. On average, participants in this study were 59 years old (SD 6.4; range 50-74 years) with 14 years of education; 63% (65/103) of the participants were non-Hispanic White and only 34% (34/103) reported being employed (part or full time). Persons with HIV were more likely to be male (79% HIV+ vs 56% HIV−; *P=*.01) with slightly less education (13.9 years HIV+ vs 15.0 years HIV−; *P=*.04). Persons with HIV had high rates of current ART use 94% (63/67), and almost all persons with HIV had an undetectable viral load 97% (60/62).

On average, participants had high survey adherence, with an average of 91% (IQR 88%-96%) of EMA surveys completed, 85% of mCWIT trials (IQR 79%-100%), and 80% of mVLT trials (IQR 71%-93%). As EMA adherence ranged from 57% to 100%, no participants were excluded because of low adherence to the mobile EMA. On average, participants reported engaging in a cognitively stimulating activity on 17% of the surveys (range 0%-61%). Participants reported performing a passive leisure activity on 33% of surveys (range 4%-79%), which was predominantly watching television (19% of total surveys on average). IADLs were reported in 20% of the surveys (range 0%-41%). The percentage of activity types did not significantly differ by HIV serostatus. The HIV− group performed better on the mCWIT (HIV+: 24.0 seconds on average vs HIV−: 21.6 seconds on average; *P=*.03); however, there was no significant difference in average performance on the mVLT by HIV serostatus (HIV+: 19.7 words on average vs HIV−: 21.2 words on average; *P=*.15). EMA variables and mobile cognitive testing variables by HIV serostatus are shown in Table 2.

### Same-Survey Associations: Activity as a Predictor of Same-Survey Neurocognitive Performance

Table 3 displays the linear mixed-effects models for all associations between activity reported on the EMA survey questions and same-survey mobile cognitive testing (ie, mCWIT and mVLT performance). Engagement in a cognitively stimulating activity (or *just prior* engagement if they stopped the activity to take the test) was significantly associated with taking less time to complete the mCWIT (β=−1.1; *P=*.007), whereas engagement in a passive leisure activity in the same survey as the mCWIT was significantly associated with taking longer to complete the mCWIT (β=.9; *P=*.005). Engaging in IADLs was not significantly associated with mCWIT performance. There was no between-person effect, as the percentage of all study surveys in which participants engaged in any of the activity types was not significantly associated with mCWIT performance (*P*>.05).

**Table 3:** Mixed-effects models for associations between activity and same-survey cognition.

Model			Estimate	95% CI	*P* value
**Mobile Color-Word Interference Test^a^**					
	**Model 1: Cognitively stimulating activities**				
		Cognitively stimulating activity (reference group: not doing activity)^b^	−1.117	−1.934 to −0.305	.007
		Percent cognitively stimulating activities^c^	0.009	−0.066 to 0.084	.82
	**Model 2: Passive leisure activities**				
		Passive leisure activity (reference group: not doing activity)^b^	0.942	0.294 to 1.595	.005
		Percent passive leisure activities^c^	0.032	−0.034 to 0.099	.35
	**Model 3: Instrumental activities of daily living**				
		Instrumental activities of daily living (reference group: not doing activity)^b^	−0.402	−1.170 to 0.369	.31
		Percent instrumental activities of daily living^c^	−0.004	−0.134 to 0.127	.96
**Mobile Verbal Learning Test^d^**					
	**Model 4: Cognitively stimulating activities**				
		Cognitively stimulating activity (reference group: not doing activity)^b^	0.313	−0.334 to 0.961	.34
		Percent cognitively stimulating activities^c^	0.070	0.011 to 0.129	.02
	**Model 5: Passive leisure activities**				
		Passive leisure activity (reference group: not doing activity)^b^	0.418	−0.103 to 0.937	.12
		Percent passive leisure activities^c^	-0.070	−0.123 to −0.016	.01
	**Model 6: Instrumental activities of daily living**				
		Instrumental activities of daily living (reference group: not doing activity)^b^	−0.255	−0.820 to 0.310	.38
		Percent instrumental activities of daily living^c^	0.009	−0.090 to 0.110	.86

^a^Mobile Color-Word Interference Test analyses controlled for study day, HIV status, age, and race or ethnicity.

^b^Modeled as a within-person variable.

^c^Modeled as between-person variables.

^d^Mobile Verbal Learning Test analyses controlled for study day, HIV status, and education.

The same-survey activity was not significantly related to mVLT performance within persons. Between persons, however, a higher percentage of surveys in which individuals reported having been engaged in cognitively stimulating activities was significantly associated with recalling more words on the mVLT on average (β=.07; *P=*.02). Upon further examination, participants in the top quartile of cognitively stimulating activities performed significantly better than participants in the bottom quartile (21.6 average mVLT words in top quartile of engagement in cognitively stimulating activities vs 17.8 average mVLT words in the bottom quartile of cognitively stimulating activities; *P*=.003; Cohen *d*=0.91). In contrast, reporting more passive leisure activities was significantly associated with fewer words recalled on the mVLT on average (β=−.07; *P=*.01). Similar to the findings with cognitively stimulating activities, participants in the lowest quartile for passive leisure activities performed significantly better on the mVLT than those in the top quartile (21.6 average mVLT words in the bottom quartile of engagement in passive leisure activities vs 17.9 average mVLT words in the top quartile of engagement in passive leisure activities; *P*=.004; Cohen *d*=0.87). The percentage of IADL activities was not significantly associated with mVLT performance. In follow-up analyses, including depressed mood as a time-varying covariate or excluding instances of alcohol and marijuana use in these models did not significantly impact any of the mCWIT or mVLT associations.

### Lagged Associations: Activity as a Predictor of Next-Survey Cognition

Table 4 shows all linear mixed-effects models in which activity (ie, doing activity or not at a given survey) predicts cognitive performance on the next EMA survey within the same day (ie, survey 1 activity predicting survey 2 mobile cognitive test, survey 2 activity predicting survey 3 mobile cognitive test, and survey 3 activity predicting survey 4 mobile cognitive test). There were no significant associations between activity and the mCWIT or mVLT at the next EMA survey. Including depressed mood as a time-varying covariate or excluding instances of alcohol and marijuana use did not change these results.

**Table 4:** Mixed-effects models for associations between activity and next-survey cognition.

Model			Estimate		95% CI		*P* value	
**Mobile Color-Word Interference Test^a^**								
	**Model 1: Cognitively stimulating activities**							
		Cognitively stimulating activity (reference group: not doing activity)^b^		0.510		−0.878 to 0.547		.39
	**Model 2: Passive leisure activities**							
		Passive leisure activity (reference group: not doing activity)^b^		0.449		−0.261 to 1.025		.38
	**Model 3: Instrumental activities of daily living**							
		Instrumental activities of daily living (reference group: not doing activity)^b^		−0.762		−0.230 to 1.083		.13
**Mobile Verbal Learning Test^c^**								
	**Model 4: Cognitively stimulating activities**							
		Cognitively stimulating activity (reference group: not doing activity)^b^		−0.167		−0.634 to 1.666		.65
	**Model 5: Passive leisure activities**							
		Passive leisure activity (reference group: not doing activity)^b^		0.385		−0.545 to 1.440		.24
	**Model 6:** **Instrumental activities of daily living**							
		Instrumental activities of daily living (reference group: not doing activity)^b^		−0.255		−1.757 to 0.236		.38

^a^Mobile Color-Word Interference Test analyses controlled for study day, HIV status, age, and race or ethnicity.

^b^Modeled as a within-person variable.

^c^Mobile Verbal Learning Test analyses controlled for study day, HIV status, and education.

## Discussion

### Principal Findings

This is one of the first studies to examine real-time relationships between activity reported on an EMA survey and mobile cognitive test performance in middle-aged and older adults in naturalistic environments. A total of 91% of EMA surveys were completed, and approximately 80% of mobile cognitive tests had valid data, thus demonstrating high adherence to the study protocol. In addition, this study demonstrates the utility of mobile cognitive testing for frequent monitoring of neurocognitive abilities to study the dynamic relationships between cognition and aspects of daily life.

Overall, we found that engagement in cognitively stimulating activities was associated with better executive functioning (mCWIT) and verbal learning (mVLT), whereas engagement in passive leisure activities was associated with worse executive functioning and verbal learning. In addition, these relationships did not seem to be explained by depressed mood or substance use (ie, alcohol or marijuana). Interestingly, it was the same-survey relationship (ie, reporting engagement in a passive leisure or cognitively stimulating activity on the same survey as taking the mCWIT) that was associated with executive functioning within persons. In both tests, the observed effects were small. For example, engagement in cognitively stimulating or passive leisure activities on average related to only about a one-second within-person difference on the mCWIT. Similarly, on the mVLT, there was only a one-word difference for a 15% difference in the percentage of cognitively stimulating activities or a 15% difference in passive leisure activities. It is possible that in daily life this effect is negligible; however, these minor differences may be more apparent in *real-world* tasks that require longer, sustained attention. Although few studies have examined the relationship between activity and cognition, the limited research does suggest that some activities such as cognitively stimulating activities, socializing, and physical activity can provide *cognitive boosts*; however, similar to this study, effect sizes are usually small [24,36-38]. Therefore, this study adds to these emerging findings by (1) suggesting that cognitively stimulating activities are associated with better neurocognitive performance and (2) being one of the first to suggest that passive leisure activities may be associated with worse executive functioning performance in middle-aged and older adults with and without HIV in the same time frame. Therefore, this observational study supports that there may be an association between activity and cognitive function, thus suggesting that real-time interventions should be investigated to examine if these interventions may yield clinically meaningful results.

An important point to consider is that, by design, the mobile cognitive tests were taken in nonstandardized environments, and performance may be impacted by other factors in the environment, such as ambient noise or multitasking. Although it is these very factors that may make mobile cognitive testing more ecologically valid, it is possible that specific activities may be related to a greater chance of distractions in the environment (eg, watching television and not turning it off while taking the test). Therefore, we cannot confirm the mechanisms underlying better or worse performance on the mobile cognitive tests (eg, distraction vs a neurobehavioral process affecting cognition).

Conversely, activities did not appear to have a real-time association with verbal learning within persons; rather, the best predictor of mVLT performance was simply the total percentage of activities an individual reported over the entire study period. The relationship between more sedentary or passive activities such as watching television and worse neurocognitive performance has been observed in both aging [14,39,40] and HIV studies [18,41] and may be because of a number of factors. For example, high levels of sedentary behavior have been linked to worse cardiovascular health, which is associated with worse neurocognitive functioning [42,43]. In addition, increased television time is also associated with psychological factors such as depression and social isolation, which have also been associated with worse neurocognitive functioning [44,45]. We speculate that the relationship between passive activities and worse learning could also be bidirectional, as those with worse overall neurocognitive functioning may be more likely to engage in more passive activities. We also found that reporting more cognitively stimulating activities was associated with better verbal learning on average. The relationship between cognitively engaging activities and better cognition has also been documented in both older adults and persons with HIV and is thought to be related to increased cognitive reserve that can be protective against neurocognitive aging and HIV-associated neurocognitive impairment [41,46,47].

There are many possible reasons that could contribute to the observed difference, whereby the executive functioning task was associated with the current activity within persons, whereas the learning task was associated with the overall percentage of cognitively stimulating and passive leisure activities. One possible explanation may be that the mCWIT was a timed test, whereas the mVLT was not. Therefore, we could speculate that processing speed may have been more affected by one’s surrounding environment and/or activity rather than executive functioning more specifically. In addition, it may be that the association between cognitively stimulating or passive leisure activities and executive functioning is more transient, whereas it is the accumulative effect of the different activities that is associated with verbal learning.

Finally, for both mobile cognitive tests, we did not observe any significant lagged effects such that activity on the previous survey (approximately 3-4 hours before) was not significantly related to cognition at the next time point. This suggests that these relationships may not be long lasting and/or that other activities in the interim may wash out the effect. In addition, because we chose to restrict the lagged analyses within the same day and therefore did not examine the relationship between activity the night prior and mobile cognitive testing in the morning, the lagged analyses did not examine morning cognition and thus may not reflect cognition throughout the entire day.

### Limitations

There are additional limitations to this study that should be considered when interpreting the results as well as to improve future research. First, it is possible that within each activity type, some activities may be more beneficial than others. For example, within IADLs, working on finances may be more cognitively stimulating than riding in a car or taking public transportation. Due to limited occurrences of specific activities, we were unable to examine the association with more specific activities. Future studies with larger sample sizes or longer monitoring periods are needed to address this limitation in the literature. Second, we do not have additional information on each specific activity in which there is likely variability; for example, we do not know what type of television programs were watched. Moreover, participants were only able to select 1 activity but may have been engaged in multiple activities (eg, watching television and cooking a meal); thus, forcing participants to choose the primary activity in which they were engaged. Future research may want to allow participants to select multiple activities and examine the impact of multitasking on functioning. Third, while the majority of participants had excellent adherence to the EMA surveys, it is possible that the proportions of activities are somewhat biased. For example, certain activities that require more cognitive demand or attention (eg, physical activity and socializing) may be associated with a greater likelihood of missing a survey, and therefore bias proportion of reported activities. Fourth, because of the low rate at which social activities and physical activity were endorsed, we were unable to examine these activities. Future research aimed at understanding the cognitive impact of physical and social activity may benefit from more frequent surveys, querying about all activities since the last survey (eg, as done by Granholm et al [48]), or integrating passive assessments of other behaviors (eg, continuous monitoring of physical activity via actigraphy). Finally, this study included a large percentage of persons with HIV who were relatively healthy, with high rates of ART use and viral suppression. Therefore, these findings may not be generalizable to all middle-aged and older adults or to all persons with HIV.

### Conclusions

This study demonstrates that it is possible to assess momentary fluctuations in cognition in relation to real-time activities in participants’ lived environments. This research methodology is particularly advantageous, given that it can be completed in participants’ own environments and does not require face-to-face contact. As a timely example, these ambulatory assessment methods could be used to investigate and track the neurocognitive changes in people recovering from COVID-19 [49].

Overall, the observed effects in this study were small, but they did suggest that cognitively stimulating activities just before testing were associated with better performance on mobile cognitive tests, whereas passive leisure activities were associated with worse performance. These results demonstrate that more research is needed to understand the contexts (such as environment, biological processes, or both) that drive these relationships to develop better recommendations and interventions to boost neurocognitive functioning. Digital health technologies may be particularly useful intervention tools, and these interventions may be particularly beneficial for older adults and older persons with HIV at greater risk for neurocognitive deficits than the general public. Given that this study only examined verbal learning and executive functioning, additional research should examine other neurocognitive domains and examine which neurocognitive domains may be most responsive to short-term variation in activities versus which are more responsive to the accumulative effects of different activities to inform future interventional research.

## Appendix

Multimedia Appendix 1 Neuropsychological battery by domain.

## Abbreviations

ADL: activities of daily living
ART: antiretroviral therapy
EMA: ecological momentary assessment
EMCA: ecological momentary cognitive assessment
HNRP: HIV Neurobehavioral Research Program
IADL: instrumental activities of daily living
mCWIT: mobile Color-Word Interference Test
mVLT: mobile Verbal Learning Test
NIMH: National Institute of Mental Health
OS: operating system
UCSD: University of California, San Diego
WRAT-4: Wide Range Achievement Test, fourth edition Reading Subtest

## References

[1] (2020). Statement on the Second Meeting of the International Health Regulations (2005) Emergency Committee Regarding the Outbreak of Novel Coronavirus (2019-nCoV). World Health Organization.

[2] Marcotte T, Grant I (2009). Neuropsychology of Everyday Functioning.

[3] Marcotte T, Scott JC, Kamat R, Heaton RK, Marcotte T, Grant I (2009). Neuropsychology and the prediction of everyday functioning. Neuropsychology of Everyday Functioning.

[4] Howieson D (2019). Current limitations of neuropsychological tests and assessment procedures. Clin Neuropsychol.

[5] Torous J, Kiang MV, Lorme J, Onnela J (2016). New tools for new research in psychiatry: a scalable and customizable platform to empower data driven smartphone research. JMIR Ment Health.

[6] Torous J, Myrick KJ, Rauseo-Ricupero N, Firth J (2020). Digital mental health and COVID-19: using technology today to accelerate the curve on access and quality tomorrow. JMIR Ment Health.

[7] Koo BM, Vizer LM (2019). Mobile technology for cognitive assessment of older adults: a scoping review. Innov Aging.

[8] Moore RC, Swendsen J, Depp CA (2017). Applications for self-administered mobile cognitive assessments in clinical research: a systematic review. Int J Methods Psychiatr Res.

[9] Germine L, Reinecke K, Chaytor NS (2019). Digital neuropsychology: challenges and opportunities at the intersection of science and software. Clin Neuropsychol.

[10] Moore RC, Campbell LM, Delgadillo JD, Paolillo EW, Sundermann EE, Holden J, Schweitzer P, Heaton RK, Swendsen J (2020). Smartphone-based measurement of executive function in older adults with and without HIV. Arch Clin Neuropsychol.

[11] Bouvard A, Dupuy M, Schweitzer P, Revranche M, Fatseas M, Serre F, Misdrahi D, Auriacombe M, Swendsen J (2018). Feasibility and validity of mobile cognitive testing in patients with substance use disorders and healthy controls. Am J Addict.

[12] Schweitzer P, Husky M, Allard M, Amieva H, Pérès K, Foubert-Samier A, Dartigues J, Swendsen J (2017). Feasibility and validity of mobile cognitive testing in the investigation of age-related cognitive decline. Int J Methods Psychiatr Res.

[13] Wilson RS, Segawa E, Boyle PA, Bennett DA (2012). Influence of late-life cognitive activity on cognitive health. Neurology.

[14] Bakrania K, Edwardson C, Khunti K, Bandelow S, Davies M, Yates T (2018). Associations between sedentary behaviors and cognitive function: cross-sectional and prospective findings from the UK biobank. Am J Epidemiol.

[15] Hoang TD, Reis J, Zhu N, Jacobs DR, Launer LJ, Whitmer RA, Sidney S, Yaffe K (2016). Effect of early adult patterns of physical activity and television viewing on midlife cognitive function. JAMA Psychiatry.

[16] Blasko I, Jungwirth S, Kemmler G, Weissgram S, Tragl K, Fischer P (2014). Leisure time activities and cognitive functioning in middle European population-based study. Eur Geriatr Med.

[17] Fancourt D, Steptoe A (2019). Television viewing and cognitive decline in older age: findings from the English longitudinal study of ageing. Sci Rep.

[18] Moore RC, Kaufmann CN, Rooney AS, Moore DJ, Eyler LT, Granholm E, Woods SP, Swendsen J, Heaton RK, Scott JC, Depp CA (2017). Feasibility and acceptability of ecological momentary assessment of daily functioning among older adults with HIV. Am J Geriatr Psychiatry.

[19] Verghese J, Lipton RB, Katz MJ, Hall CB, Derby CA, Kuslansky G, Ambrose AF, Sliwinski M, Buschke H (2003). Leisure activities and the risk of dementia in the elderly. N Engl J Med.

[20] Fazeli PL, Woods SP, Heaton RK, Umlauf A, Gouaux B, Rosario D, Moore RC, Grant I, Moore DJ, HNRP Group (2014). An active lifestyle is associated with better neurocognitive functioning in adults living with HIV infection. J Neurovirol.

[21] Farias ST, Mungas D, Jagust W (2005). Degree of discrepancy between self and other-reported everyday functioning by cognitive status: dementia, mild cognitive impairment, and healthy elders. Int J Geriatr Psychiatry.

[22] Thames AD, Becker BW, Marcotte TD, Hines LJ, Foley JM, Ramezani A, Singer EJ, Castellon SA, Heaton RK, Hinkin CH (2011). Depression, cognition, and self-appraisal of functional abilities in HIV: an examination of subjective appraisal versus objective performance. Clin Neuropsychol.

[23] Harvey PD, Velligan DI, Bellack AS (2007). Performance-based measures of functional skills: usefulness in clinical treatment studies. Schizophr Bull.

[24] Allard M, Husky M, Catheline G, Pelletier A, Dilharreguy B, Amieva H, Pérès K, Foubert-Samier A, Dartigues J, Swendsen J (2014). Mobile technologies in the early detection of cognitive decline. PLoS One.

[25] Plassman BL, Langa KM, Fisher GG, Heeringa SG, Weir DR, Ofstedal MB, Burke JR, Hurd MD, Potter GG, Rodgers WL, Steffens DC, McArdle JJ, Willis RJ, Wallace RB (2008). Prevalence of cognitive impairment without dementia in the United States. Ann Intern Med.

[26] Makinson A, Dubois J, Eymard-Duvernay S, Leclercq P, Zaegel-Faucher O, Bernard L, Vassallo M, Barbuat C, Gény C, Thouvenot E, Costagliola D, Ozguler A, Zins M, Simony M, Reynes J, Berr C (2020). Increased prevalence of neurocognitive impairment in aging people living with human immunodeficiency virus: the ANRS EP58 hand 55-70 study. Clin Infect Dis.

[27] Heaton RK, Clifford DB, Franklin DR, Woods SP, Ake C, Vaida F, Ellis RJ, Letendre SL, Marcotte TD, Atkinson JH, Rivera-Mindt M, Vigil OR, Taylor MJ, Collier AC, Marra CM, Gelman BB, McArthur JC, Morgello S, Simpson DM, McCutchan JA, Abramson I, Gamst A, Fennema-Notestine C, Jernigan TL, Wong J, Grant I, CHARTER Group (2010). HIV-associated neurocognitive disorders persist in the era of potent antiretroviral therapy: CHARTER study. Neurology.

[28] Jeste DV, Palmer BW, Appelbaum PS, Golshan S, Glorioso D, Dunn LB, Kim K, Meeks T, Kraemer HC (2007). A new brief instrument for assessing decisional capacity for clinical research. Arch Gen Psychiatry.

[29] World Health Organization (1997). Composite International Diagnostic Interview (CIDI, version 2.1).

[30] Cysique LA, Franklin D, Abramson I, Ellis RJ, Letendre S, Collier A, Clifford D, Gelman B, McArthur J, Morgello S, Simpson D, McCutchan JA, Grant I, Heaton RK, CHARTER Group, HNRC Group (2011). Normative data and validation of a regression based summary score for assessing meaningful neuropsychological change. J Clin Exp Neuropsychol.

[31] Carey CL, Woods SP, Gonzalez R, Conover E, Marcotte TD, Grant I, Heaton RK, HNRC Group (2004). Predictive validity of global deficit scores in detecting neuropsychological impairment in HIV infection. J Clin Exp Neuropsychol.

[32] Wilkinson GS, Robertson GJ (2006). Wide Range Achievement Test (WRAT4).

[33] Hinkin CH, Castellon SA, Hardy DJ, Granholm E, Siegle G (1999). Computerized and traditional stroop task dysfunction in HIV-1 infection. Neuropsychology.

[34] Iudicello JE, Woods SP, Deutsch R, Grant I, HIV Neurobehavioral Research Program Hnrp Group (2012). Combined effects of aging and HIV infection on semantic verbal fluency: a view of the cortical hypothesis through the lens of clustering and switching. J Clin Exp Neuropsychol.

[35] Benedict RH, Schretlen D, Groninger L, Brandt J (1998). Hopkins verbal learning test – revised: normative data and analysis of inter-form and test-retest reliability. Clin Neuropsychol.

[36] Ybarra O, Winkielman P, Yeh I, Burnstein E, Kavanagh L (2010). Friends (and sometimes enemies) with cognitive benefits. Soc Psychol Pers Sci.

[37] Ybarra O, Burnstein E, Winkielman P, Keller MC, Manis M, Chan E, Rodriguez J (2008). Mental exercising through simple socializing: social interaction promotes general cognitive functioning. Pers Soc Psychol Bull.

[38] Chang YK, Labban JD, Gapin JI, Etnier JL (2012). The effects of acute exercise on cognitive performance: a meta-analysis. Brain Res.

[39] Kesse-Guyot E, Charreire H, Andreeva VA, Touvier M, Hercberg S, Galan P, Oppert J (2012). Cross-sectional and longitudinal associations of different sedentary behaviors with cognitive performance in older adults. PLoS One.

[40] Hamer M, Stamatakis E (2014). Prospective study of sedentary behavior, risk of depression, and cognitive impairment. Med Sci Sports Exerc.

[41] Milanini B, Ciccarelli N, Fabbiani M, Limiti S, Grima P, Rossetti B, Visconti E, Tamburrini E, Cauda R, di Giambenedetto S (2016). Cognitive reserve and neuropsychological functioning in older HIV-infected people. J Neurovirol.

[42] Wilmot EG, Edwardson CL, Achana FA, Davies MJ, Gorely T, Gray LJ, Khunti K, Yates T, Biddle SJ (2012). Sedentary time in adults and the association with diabetes, cardiovascular disease and death: systematic review and meta-analysis. Diabetologia.

[43] Biswas A, Oh PI, Faulkner GE, Bajaj RR, Silver MA, Mitchell MS, Alter DA (2015). Sedentary time and its association with risk for disease incidence, mortality, and hospitalization in adults: a systematic review and meta-analysis. Ann Intern Med.

[44] Boss L, Kang D, Branson S (2015). Loneliness and cognitive function in the older adult: a systematic review. Int Psychogeriatr.

[45] Teychenne M, Ball K, Salmon J (2010). Sedentary behavior and depression among adults: a review. Int J Behav Med.

[46] Yates LA, Ziser S, Spector A, Orrell M (2016). Cognitive leisure activities and future risk of cognitive impairment and dementia: systematic review and meta-analysis. Int Psychogeriatr.

[47] Stern Y (2012). Cognitive reserve in ageing and Alzheimer's disease. Lancet Neurol.

[48] Granholm E, Holden JL, Mikhael T, Link PC, Swendsen J, Depp C, Moore RC, Harvey PD (2020). What do people with schizophrenia do all day? Ecological momentary assessment of real-world functioning in schizophrenia. Schizophr Bull.

[49] Holmes EA, O'Connor RC, Perry VH, Tracey I, Wessely S, Arseneault L, Ballard C, Christensen H, Cohen Silver R, Everall I, Ford T, John A, Kabir T, King K, Madan I, Michie S, Przybylski AK, Shafran R, Sweeney A, Worthman CM, Yardley L, Cowan K, Cope C, Hotopf M, Bullmore E (2020). Multidisciplinary research priorities for the COVID-19 pandemic: a call for action for mental health science. Lancet Psychiatry.

