# Supplementary material for: Daily Activities Related to Mobile Cognitive Performance in Middle-Aged and Older Adults: An Ecological Momentary Cognitive Assessment Study
Source: JMIR Mhealth Uhealth. 2020 Sep 24;8(9):e19579. doi: 10.2196/19579 (PMC7545331; doi:10.2196/19579)
Supplement: Multimedia Appendix 1 [file mhealth_v8i9e19579_app1.docx]

Multimedia Appendix 1. Neuropsychological Battery by Domain

| **Verbal Fluency** | **Speed of Information Processing** |
| --- | --- |
| Controlled Oral Word Association Test (FAS) | WAIS-III Digit Symbol |
| Category Fluency Test (“animals” and “actions”) | WAIS-III Symbol Search |
| **Executive Functions** | Trail Making Test Part A |
| Wisconsin Card Sorting Test (computerized 64-item version) | Stroop Color and Word Test (color trial) |
| Trail Making Test Part B | **Working Memory** |
| Stroop Color and Word Test (interference trial) | WAIS-III Letter-Number Sequencing |
| **Learning and Memory (2 domains)** | Paced Auditory Serial Addition Task |
| Hopkins Verbal Learning Test-Revised | **Complex Motor Skills** |
| Brief Visuospatial Memory Test-Revised | Grooved Pegboard Test (dominant and non-dominant hands) |

*Note.* WAIS-III = Wechsler Adult Intelligence Test 3^rd^ Edition; WRAT-4 = Wide Range Achievement Test 4^th^ Edition
